# Characterization of *DWARF14* Genes in *Populus*

**DOI:** 10.1038/srep21593

**Published:** 2016-02-15

**Authors:** Kaijie Zheng, Xiaoping Wang, Deborah A. Weighill, Hao-Bo Guo, Meng Xie, Yongil Yang, Jun Yang, Shucai Wang, Daniel A. Jacobson, Hong Guo, Wellington Muchero, Gerald A. Tuskan, Jin-Gui Chen

**Affiliations:** 1 Biosciences Division, Oak Ridge National Laboratory, Oak Ridge, TN 37831, USA; 2 Key Laboratory of Molecular Epigenetics of Ministry of Education, Institute of Genetics and Cytology, Northeast Normal University, Changchun 130024, China; 3 Department of Biochemistry and Cellular and Molecular Biology, University of Tennessee Knoxville, TN 37996, USA; 4 National Key Laboratory of Plant Molecular Genetics, Institute of Plant Physiology and Ecology, Shanghai Institutes for Biological Sciences, Chinese Academy of Sciences, Shanghai 200032, China; 5 Bredesen Center for Interdisciplinary Research and Graduate Education, University of Tennessee, Knoxville, TN 37996, USA; 6 Genome Science and Technology Program, University of Tennessee, Knoxville, TN 37996, USA

## Abstract

Strigolactones are a new class of plant hormones regulating shoot branching and symbiotic interactions with arbuscular mycorrhizal fungi. Studies of branching mutants in herbaceous plants have identified several key genes involved in strigolactone biosynthesis or signaling. The strigolactone signal is perceived by a member of the α/β-fold hydrolase superfamily, known as DWARF14 (D14). However, little is known about D14 genes in the woody perennial plants. Here we report the identification of D14 homologs in the model woody plant *Populus trichocarpa*. We showed that there are two D14 homologs in *P. trichocarpa*, designated as PtD14a and PtD14b that are over 95% similar at the amino acid level. Expression analysis indicated that the transcript level of *PtD14a* is generally more abundant than that of *PtD14b*. However, only *PtD14a* was able to complement Arabidopsis *d14* mutants, suggesting that PtD14a is the functional D14 ortholog. Amino acid alignment and structural modeling revealed substitutions of several highly conserved amino acids in the PtD14b protein including a phenylalanine near the catalytic triad of D14 proteins. This study lays a foundation for further characterization of strigolactone pathway and its functions in the woody perennial plants.

Strigolactones (SLs) are a new class of plant hormones regulating shoot branching^1^,^2^ and symbiotic interactions with arbuscular mycorrhizal fungi^3^,^4^. In addition, SLs regulate many other processes in plant growth and development including primary root growth, lateral root formation, adventitious root formation, root hair development, seed germination, photomorphogenesis and nodulation (reviewed in references^5^,^6^,^7^,^8^,^9^,^10^,^11^), protonema branching in moss^12^, as well as responses to stresses^13^ and nutrient deficiency (reviewed in reference^8^). In the last decade, great progresses have been made to identify genes regulating the biosynthesis and signaling of SLs, in particular, by analyzing branching mutants in Arabidopsis, pea, rice and petunia (reviewed in references^14^,^15^,^16^,^17^,^18^,^19^,^20^,^21^,^22^,^23^). It has been demonstrated that the biosynthesis of SLs is mainly regulated by two carotenoid cleavage dioxygenases, CCD7 [encoded by *MORE AXILLARY GROWTH 3* (*MAX3*) gene in Arabidopsis] and CCD8 (encoded by *MAX4* gene in Arabidopsis), one cytochrome P450 monooxygenase (encoded by *MAX1* gene in Arabidopsis) and one novel iron-containing protein (DWARF27)^24^,^25^,^26^,^27^,^28^,^29^. Loss-of-function mutations in each of these genes resulted in increased number of branches.

In Arabidopsis, the signaling of SLs is mainly regulated by MAX2^24^, an F-box leucine-rich protein, and DWARF14 (D14), a member of the α/β-fold hydrolase superfamily^30^,^31^,^32^,^33^. Several lines of evidence support that D14 functions as a receptor for SLs. Genetic analysis indicated that loss-of-function mutations in *D14* resulted in increased number of branches and that *d14* mutants are insensitive to SLs^30^,^31^. At the biochemical level, D14 can directly bind SLs^32^. Furthermore, SL-stimulated degradation of downstream targets depends on D14^34^,^35^ and D14 interacts with MAX2 in an SL concentration-dependent manner to regulate branching^32^,^36^. The degradation of D14 protein itself is also SL-induced and MAX2-dependent^37^.

To date, no D14 orthologs have been reported in woody plants. Because shoot branching plays an important role determining photosynthetic light use efficiency and biomass yield, study on strigolactone pathways helps inform genetic improvement of woody plants to increase biomass production in the forestry, horticultural and emerging biofuels industries. Therefore, we investigate D14 orthologs in the model woody plant, *Populus trichocarpa*.

## Results

### D14 sequence homologs in *Populus trichocarpa*

**Figure 1:**
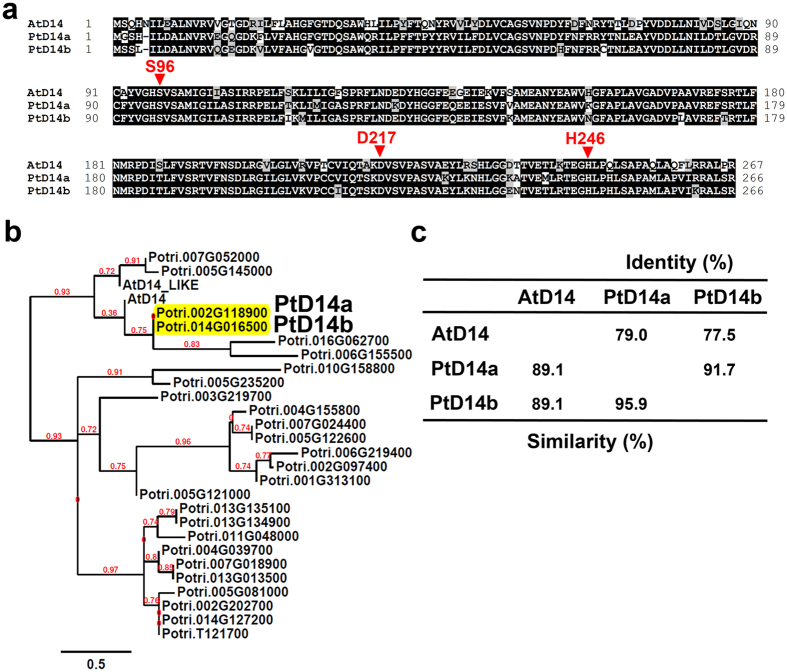
Bioinformatics analysis of D14 proteins from *Populus* and Arabidopsis. (**a**) Amino acid sequence alignment of Arabidopsis D14 with its *Populus* sequence homologs. The catalytic triad Ser96-His246-Asp217 of PtD14a/b proteins is indicated by arrow heads on top of the amino acids. (**b**) Phylogenetic analysis of *Populus* sequence homologues of *Arabidopsis* D14 and D14L proteins. (**c**) Percentage of amino acid similarity and identity among Arabidopsis D14 and its *Populus* sequence homologs.

In order to identify sequence homologs of D14 in *Populus trichocarpa* (hereafter referred to as *Populus*), we used amino acid sequence of Arabidopsis D14 protein (encoded by locus At3g03990) as a template to perform protein homology search in the fully-sequenced *Populus* genome using the “Protein Homologs” search tool at Phytozome (www.phytozome.net)^38^. The search identified two closest sequence homologues encoded by loci Potri.002G118900 and Potri.014G016500, designated as PtD14a and PtD14b, respectively (Fig. 1, Table 1). PtD14a has 89.1% similarity and 79% identity and PtD14b has 89.1% similarity and 77.5% identity with AtD14 at the amino acid level. PtD14a and PtD14b share 95.9% similarity and 91.7% identity with each other at the amino acid level. Based on high amino acid sequence identity and similarity and their syntenic positions on chromosomes 2 and 14, it appears that PtD14a and PtD14b are paralogs resulting from the Salicoid whole genome duplication event^39^. The number of amino acids of PtD14a (266 aa) and PtD14b (266 aa) are almost identical to that of AtD14 (267 aa) (Fig. 1a). The catalytic triad Ser97-His247-Asp218 of AtD14 (Ser96-His246-Asp217 of PtD14a and PtD14b) is completely conserved in PtD14 proteins (Fig. 1a). No other *Populus* proteins showed more than 77% similarity or 51% identity with AtD14 at the amino acid level (Table 1). The two other sequence homologs present in the same phylogenetic cluster of PtD14a and PtD14b are encoded by loci Potri.016G062700 and Potri.006G155500. However, these two proteins only showed 43.1% and 41.4% identity with AtD14 at the amino acid level and are much larger in size (i.e., 276aa and 278aa) (Figure S1). Therefore, we hypothesized that compared to PtD14a and PtD14b, proteins encoded by loci Potri.016G062700 and Potri.006G155500 have low probability being *Populus* orthologs of AtD14.

It should be noted that there is an AtD14-LIKE (AtD14L)/KARRIKIN INSENSITIVE 2 (KAI2) protein in Arabidopsis encoded by locus At4g37470. AtD14L is 75.9% similar and 51% identical to AtD14 at the amino acid level (Table 1). Genetic studies indicated that AtD14L mediates the response to karrikins, but not to strigolactones^31^. A phylogenetic study also indicated that AtD14 and AtD14L are members of distinct phylogenetic clades among land plants^31^. Because AtD14L is closely related to AtD14 in terms of amino acid sequence, we also included *Populus* sequence homologs of AtD14L in our analysis to further validate that we have identified true sequence homologs of AtD14, but not of AtD14L, in *Populus*. When the amino acid sequence AtD14L was used as a template to perform protein homology search in the *Populus* genome using the “Protein Homologs” search tool at Phytozome, two sequence homologs were identified (Potri.007G05200 and Potri.005G14500) (Table 1). Phylogenetic analysis indicated that Potri.007G05200 and Potri.005G14500 are clustered together with AtD14L, whereas PtD14a and PtD14b are clustered with AtD14 (Fig. 1b, Figures S2 and S3). This analysis supports our hypothesis that Potri.002G118900 and Potri.014G016500 are likely the *Populus* orthologs of AtD14. Therefore, we focused our studies on these two genes.

### Expression patterns of *PtD14* genes

**Figure 2:**
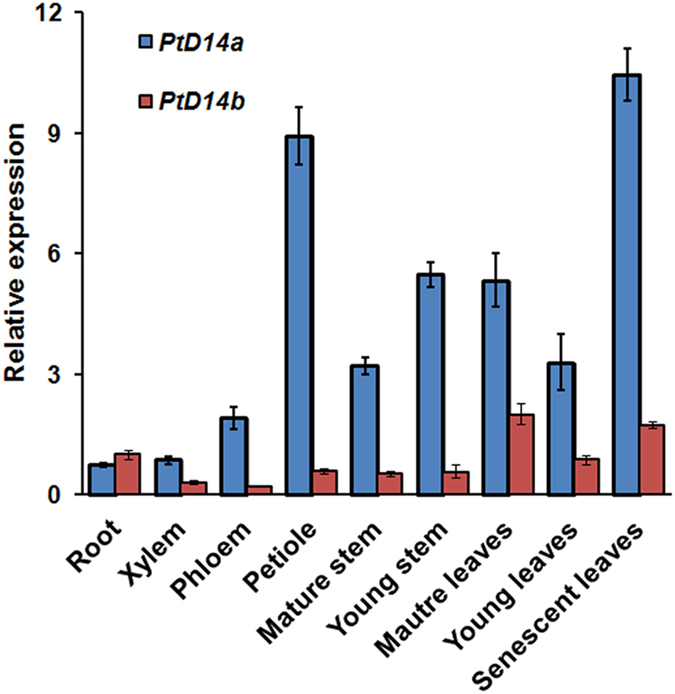
Expression of *Populus* D14 genes across various tissues and organs. Shown are quantitative RT-PCR data using *PtD14* gene-specific primers. PCR amplification of *Populus UBIQUITIN C* (*UBC*) was used as a control. The expression value of *PtD14b* in root was set at 1

In order to gain insights into the function of *PtD14* genes, we examined the expression patterns of *PtD14* genes across nine tissues/organs by using quantitative RT-PCR. The transcripts of *PtD14a* and *PtD14b* were detected in all tissues and organs examined. However, the transcript of *PtD14a* is generally more abundant than that of *PtD14b* in these tissues (Fig. 2). These results are largely consistent with the RNAseq data from the *Populus* gene atlas study in Phytozome though tissue and organ types examined were not identical between these two datasets (Figure S4).

### Co-expression patterns of *PtD14* genes

**Figure 3:**
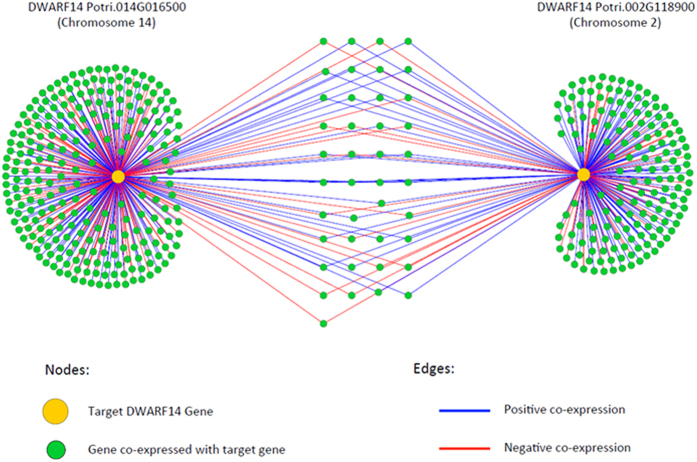
Co-expression networks of *Populus* D14 genes. Pearson correlation coefficient ≥0.95 was used to select co-expressed genes. Both positive and negative co-expressions are shown.

In order to explore the potential functional difference between *PtD14a* and *PtD14b*, we generated their co-expression networks. Using Pearson correlation coefficient ≥0.95 to identify genes highly co-expressed with *PtD14a* or *PtD14b*, we identified a total of 200 genes co-expressed (both positive and negative co-expressions) with *PtD14a* and a total of 291 genes co-expressed with *PtD14b* (Table S1). Interestingly, only less than 10% of these genes are co-expressed with both *PtD14a* and *PtD14b* (Fig. 3, Table S1). In addition, in all cases where *PtD14a* or *PtD14b* shared co-expressed genes the direction of the correction (i.e., positive or negative) was always the same. Because co-expression networks are implications of connected purpose, our results suggested that the functions of *PtD14a* and *PtD14b* may have been evolved differently post-Salicoid duplication.

### Genetic complementation of Arabidopsis *d14* mutants

**Figure 4:**
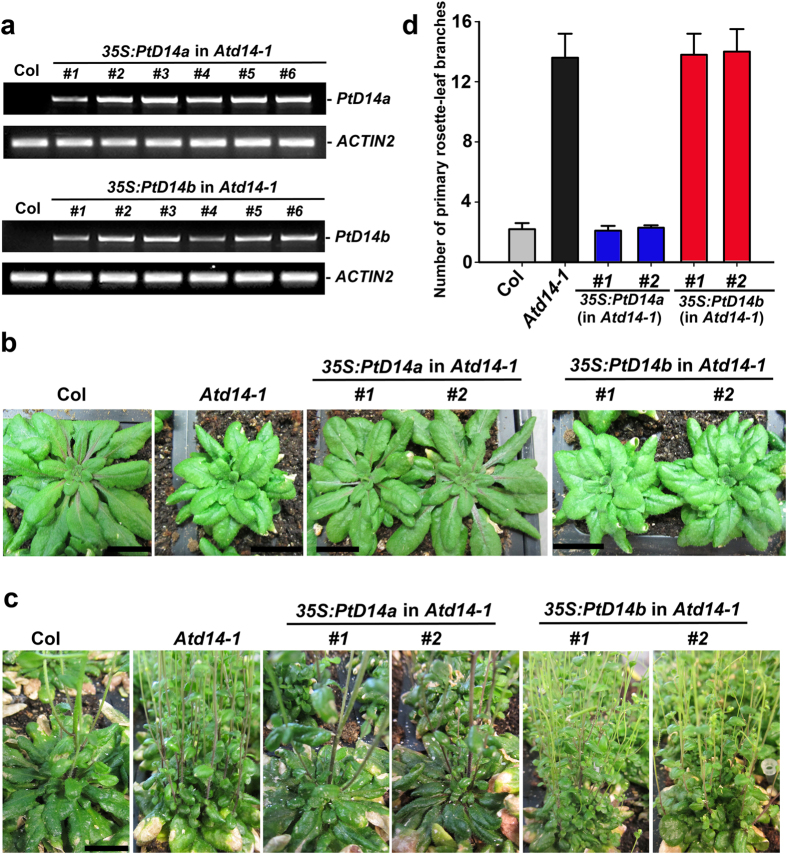
Genetic complementation of *Arabidopsis d14* mutant with *Populus D14* genes. (**a**) RT-PCR analysis of *35S:PtD14a* and *35S:PtD14b* transgenic lines. PCR amplification of *ACTIN2* was used as a control. (**b**) Rosette phenotypes of *35S:PtD14a* and *35S:PtD14b* transgenic lines at the vegetative growth stage. Two independent transgenic lines from each transformation are shown. Scale bar, 1 cm. (**c**) Shoot branching phenotypes of *35S:PtD14a* and *35S:PtD14b* transgenic lines at the bolting stage. Two independent transgenic lines from each transformation are shown. Scale bar, 1 cm. (**d)** Number of primary rosette-leaf branches. Shown are the branch numbers from two independent transgenic lines from each transformation. The branching phenotypes were scored from T2 transgenic lines. Shown are averages of a minimum of 10 plants ± S.E.

To test whether *PtD14a* or *PtD14b* or both are the functional orthologs of *D14* and to test whether *PtD14a* and *PtD14b* may involve alternative functions, separate from or complementary to *AtD14*, we performed a genetic complementation experiment using Arabidopsis *d14* mutants. The expression of each *PtD14* gene was driven by the constitutive *35S* promoter. A minimum of 20 independent transgenic lines were obtained from each transformation and six independent transgenic lines were selected for further analysis. RT-PCR analysis indicated that *PtD14* transgene was expressed in each of these independent transgenic lines (Fig. 4a). At the vegetative growth stage, *Atd14* mutants had smaller rosette size with smaller leaves compared to wild-type Col-0. These phenotypes were completely rescued to wild-type in *35S:PtD14a* transgenic lines, but not in *35S:PtD14b* transgenic lines (Fig. 4b). A more characteristic phenotype of *Atd14* mutant is its increased number of primary rosette-leaf branches. This branching phenotype was also completely rescued to wild-type in *35S:PtD14a* transgenic lines, but not in *35S:PtD14b* transgenic lines (Fig. 4c,d). Under our growth conditions, Col-0 wild-type produced about two primary rosette-leaf branches whereas *Atd14-1* mutants produced about 13 primary rosette-leaf branches. The number of primary rosette-leaf branches in the *35S:PtD14a* transgenic lines was reverted to typical wild-type whereas the number of primary rosette-leaf branches in the *35S:PtD14b* transgenic lines was near identical to the *Atd14-1* mutants. These results indicated that PtD14a, but not PtD14b, is the *Arabidopsis* D14 functional ortholog.

### Structural modeling of PtD14 proteins

**Figure 5:**
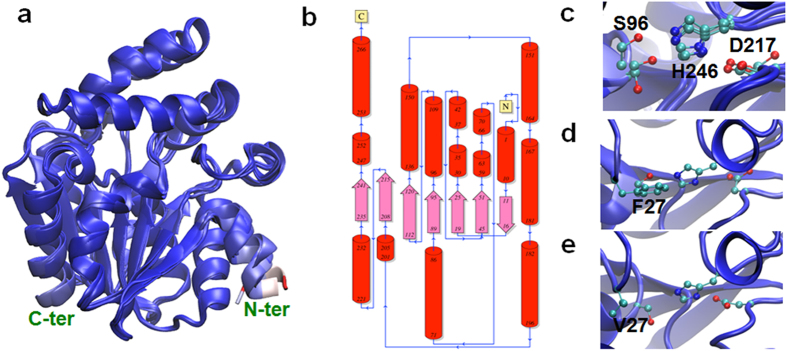
Structural models of PtD14 proteins. (**a**) Superimposed structures of the three models (AtD14, PtD14a and PtD14b) colored by a Blue-White-Red model: blue for structurally conserved, red for deviated and white for between blue and red. Only the N-termini have relatively high deviations. (**b**) 2D architecture of the PtD14a protein drawn by PDBSum. The 2D architecture of PtD14b is identical to that of PtD14a. (**c**) The active site with the catalytic triad of the three models; residue numbers are from the PtD14a/b. (**d**) The active site of PtD14a with F27. (**e**) The active site of PtD14b with V27.

Amino acid sequence comparison indicated that PtD14a and PtD14b are 95.9% similar and 91.7% identical to each other at the amino acid level (Fig. 1, Table 1). However, co-expression analysis and genetic complementation study suggested that PtD14a and PtD14b may function differently (Figs 3 and 4). To investigate the potential functional difference at the amino acid level, we performed structural modeling of PtD14 proteins with I-TASSER^40^,^41^. Crystallographic structure of AtD14 [PDB entry 4IH4^42^] contains residues I6 to P266, which lacks the N-terminal residues M1 to N5 and the C-terminal residue R267. Therefore, to minimize the systematic errors, the structural model of AtD14 was reconstructed using its full-length sequence from M1 to R267. Not surprisingly, all models of PtD14a, PtD14b and AtD14 superimposed very well to each other, as well as to the AtD14 crystal structure. Only small structural deviations were observed in the N-termini (Fig. 5a and Figure S5), which may also explain the absence of the N-terminal residues in the crystallographic structure. Interestingly, all three models share the same 2D architecture (Fig. 5b). However, a closer examination of amino acids near the catalytic center (i.e., catalytic triad, S96-H246-D217 of the PtD14 proteins) revealed a compelling difference. Both AtD14 and PtD14a have a phenylalanine (F27 of PtD14a and F28 of AtD14) located in the vicinity of the nucleophile serine residue (S96) of the catalytic triad. In PtD14b, however, an F27V replacement resulted in the separation of V27 from S96 (Fig. 5c–e), which presumably affects ligand binding stability as further discussed below.

**Figure 6:**
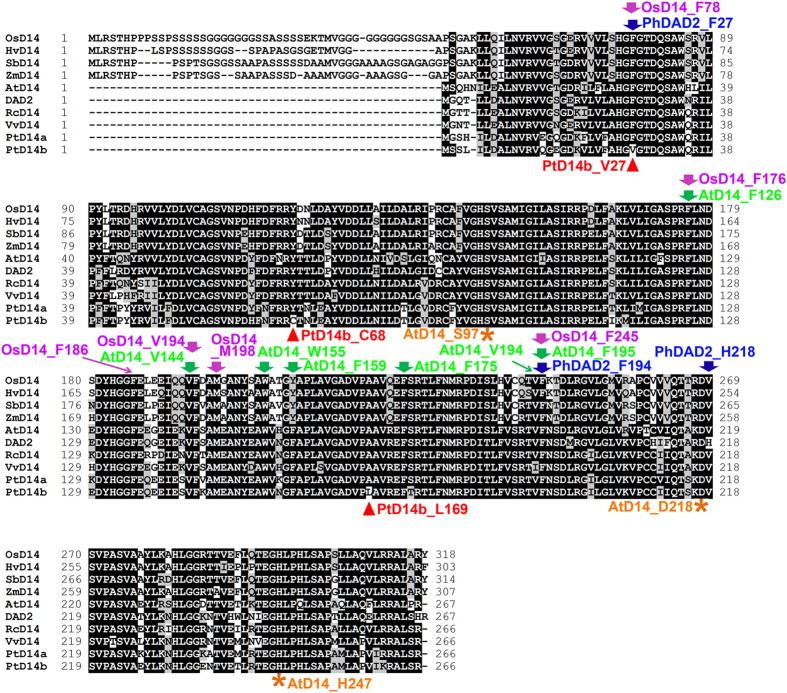
Amino acid sequence alignment of D14 proteins and D14 orthologs in monocots (rice, barley, sorghum and maize) and dicots (Arabidopsis, petunia, castor bean, grape and poplar). The catalytic triad Ser97-His247-Asp218 of AtD14 is indicated by asterisks on bottom of the amino acids. The amino acids of F126, V144, W155, F159, F175, V194 and F195 of the AtD14 protein, the amino acids of F78, F176, F186, V194, M198 and F245 of the OsD14 protein and the amino acids of F27, F194 and H218 of PhDAD2 are indicated by arrows on the top of amino acids. These amino acids have been shown to be important for the structure and function of these proteins in the crystal structure studies. Amino acids that are invariants in PtD14a and other D14 homologs but are variants in PtD14b are indicated by arrow heads on the bottom of amino acids.

In order to investigate whether phenylalanine at this position is conserved in D14 sequence homologs in other plant species, we selected D14 sequence homologs from four representative monocots (i.e., rice, barley, sorghum and maize) and five representative dicots (i.e., Arabidopsis, petunia, castor bean, grape and poplar). Amino acid alignment of these D14 sequence homologs indicated that phenylalanine at this position is completely conserved in all D14 orthologs except PtD14b (Fig. 6). Moreover, analysis of crystal structures of D14 protein in Arabidopsis, rice and petunia supports that phenylalanine at this position (F78 in OsD14 and F27 in PhDAD2) is critical for the structure and function of D14 proteins^32^,^42^,^43^. F78 in OsD14 is a part of the entrance of the active site pocket and also a part of the conserved Gly-Phe-Gly segment that stabilizes ligand binding^43^. Similarly, F27 in PhDAD2 has its main chain slightly moved away from A96 of the PhDAD2S96A mutant protein and its side chain shows weak discontinuous electron density, suggestive of partially disordered^32^. Hamiaux *et al.* (2012) have suggested the flexibility of F27 inside the DAD2S96A mutant’s cavity may interfere with binding of GR24^32^. In addition, F27 was also one of the seven phenylalanine residues surrounding the internal cavity. Before the crystal structures of D14 were available, Gaiji *et al.* (2012), using a computational-based structure study, proposed the phenylalanine at this position (F78 in OsD14) as one of the key amino acids involved in ligand binding^44^. Results from these studies reinforced the importance of the phenylalanine reside at this position for its function.

It should be noted that amino acid alignment of D14 sequence homologs from representative species of monocots and dicots also identified two other amino acids that are invariants in PtD14a and other D14 homologs but are variants in PtD14b. These are cysteine68 (C68) and leucine (L169) of PtD14b (Fig. 6). Structural modeling indicated that both of these residues are located at the protein periphery and distal to the catalytic triad (data not shown), implying that C68 and L169 have less impact on the structure or activity of PtD14b protein.

### Biochemical analysis of PtD14 proteins

**Figure 7:**
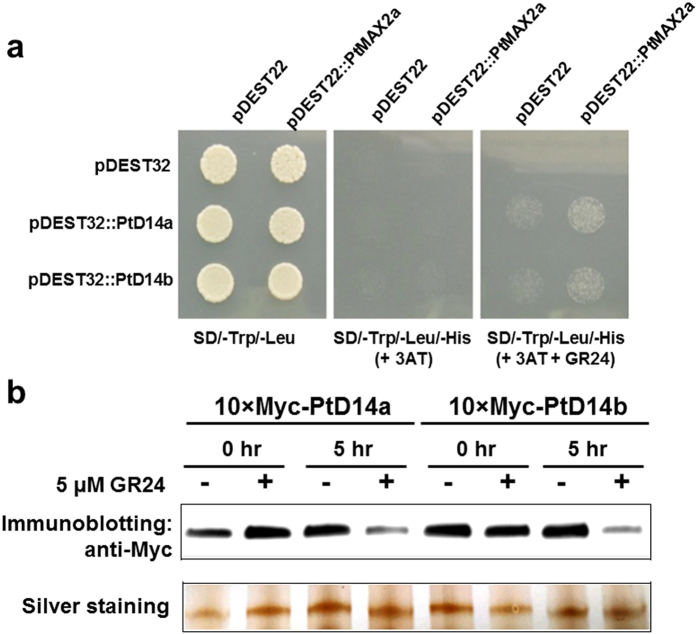
Biochemical analysis of PtD14 proteins. **(a)** Yeast two-hybrid assay. PtD14a and PtD14b in the pDEST32 destination vector were used as baits. PtMAX2a in the pDEST22 destination vector was used as prey. Co-transformation of pDEST32 and pDEST22 empty vectors and co-transformations of the empty vector with pDEST vectors containing PtD14a, PtD14b or PtMAX2a were used as negative controls. Yeast cells grown on SD plate deficient of Trytophane and Leucine (SD/-Trp/-Leu) indicate correctly co-transformation. Yeast cells grown on SD plate deficient of Tryptophan, Leucine, and Histidine (SD/-Trp/-Leu/-His) supplemented with 5 mM 3-Amino-1,2,4-triazole (3AT) and with or without 5 μM GR24 indicate the interactions between PtD14a/b and PtMAX2a. **(b)** Degradation of PtD14 proteins. Myc-tagged PtD14a and PtD14b proteins were transiently expressed in the *Populus* leaf mesophyll protoplasts. GR24 was added into the transfected protoplast suspension to a final concentration of 5 μM and incubated for another 5 hr. Myc-tagged PtD14 proteins were detected by using anti-Myc antibody. One protein band existing in all samples (~80 kDa) was used to illustrate equal protein loading.

To examine whether PtD14a and PtD14b proteins may behave differently at the biochemical level pertaining to the SL pathways, we applied two well established biochemical assays. In the first assay, we examined the interactions between PtD14a/b and PtMAX2a, one of the two *Populus* MAX2 orthologs^45^, using the yeast two-hybrid system. In this system, D14 interaction with MAX2 was shown to be GR24-dependent, as had been demonstrated for petunia D14 and MAX2 orthologs^32^. As expected, neither PtD14a nor PtD14b interacted with PtMAX2a in the absence of GR24 (Fig. 7a). In the presence of GR24, both PtD14a and PtD14b interacted with PtMAX2a (Fig. 7a), indicating that PtD14a and PtD14b do not differ in this biochemical property. In the second assay, we examined the degradation of PtD14a and PtD14b proteins by GR24. It has been previously established in Arabidopsis that the degradation of D14 protein is induced by GR24, which provides a negative feedback loop in SL signaling^37^. In our assays, Myc-tagged PtD14a and PtD14b proteins were transiently expressed using the *Populus* leaf mesophyll protoplast system^46^, and anti-Myc antibodies were used for immunoblotting. As shown in Fig. 7b, GR24 at 5 μM was sufficient to induce the degradation of both PtD14a and PtD14b proteins after 5 hr treatment, indicating that PtD14a and PtD14b also do not differ in this biochemical property.

## Discussion

D14 has previously been shown to function as a receptor for SLs and the characterization of D14 and its orthologs has been reported in Arabidopsis, rice and petunia. Here we report the identification and characterization of D14 orthologs in the model woody plant *Populus*.

Two sequence homologs of D14 were identified in *Populus* (Fig. 1, Figure S2, Table 1). The expression patterns between these two homologs are similar but *PtD14a* was expressed at higher levels than *PtD14b* in most tissues and organs examined (Fig. 2, Figure S4). The expression analysis suggested that PtD14a is likely a predominant form of *D14* in *Populus*. Remarkably, although PtD14a and PtD14b are highly similar at the amino acid level (i.e., 95.9% similarity and 91.7% identity), only *PtD14a*, but not *PtD14b*, was able to fully complement Arabidopsis *d14* mutants when their expression was driven by the constitutive *35S* promoter (Fig. 4). These results indicated that PtD14a is the functional *Populus* ortholog of D14 and that differences at the amino acid level are likely responsible for PtD14a and PtD14b’s functional difference. Protein structural modeling and amino acid alignment revealed substitutions of several highly conserved amino acids including a phenylalanine near the catalytic triad of the PtD14b protein (Figs 5 and 6). However, both PtD14a and PtD14b interacted with PtMAX2a in a GR24-dependent manner and GR24 induced the degradation of both PtD14a and PtD14b proteins (Fig. 7), indicating that PtD14a and PtD14b proteins may behave similarly at the biochemical level. It is intriguing that how these two proteins could differ genetically but behave similarly at the biochemical level. One speculation was that these two biochemical assays (yeast two-hybrid assay and GR24-induced protein degradation assay) may not have sufficient sensitivities to detect subtle differences. It is also possible that the downstream cascades triggered by PtD14a and PtD14b may differ, leading to different consequences. The latter possibility may be partially explained by the small number of shared co-expressed genes between *PtD14a* and *PtD14b* (Fig. 3). With limited information available for SL signaling pathway in *Populus*, full assessment of this possibility requires further investigation.

*PtD14a* and *PtD14b* are located in chromosome 2 and chromosome 14, respectively. These two chromosomes possess high rates of gene duplication events, likely derived from whole genome duplication event^39^. Genome duplication often results in diversification of gene function^47^. It is likely that PtD14b is the paralog of PtD14a and has subsequently accumulated novel mutations that affect its function and co-expression gene networks. The genetic complementation with Arabidopsis *d14* mutants suggested PtD14b has diversified from its original function of regulating shoot branching. It remains unknown, however, what new functions *PtD14b* has evolved. This would require further genetic studies. For example, analyzing *Populus* transgenic plants over- or under-expressing each of these genes may provide further insights into the function of these genes.

Previously, we have identified orthologs of each of four founding members of strigolactone biosynthesis and signaling pathway genes, namely *MAX1*, *MAX2*, *MAX3* and *MAX4*, in *Populus*^45^. In combination with the identification of D14 ortholog in *Populus*, a strigolactone biosynthesis and signaling pathway in woody perennial plants, similar to those in herbaceous species, is emerging. Future studies should focus on the functional characterization of these SL pathway genes (i.e., analyzing transgenic lines in *Populus*), the detection of SL molecules and the determination of the physiological roles of SLs in woody perennials. Because shoot branching plays an important role determining photosynthetic light use efficiency and biomass yield, the study of SLs has the potential to increase biomass production in the forestry, horticultural and emerging biofuels industries.

## Materials and Methods

### Protein homolog search, sequence alignment and phylogenetic analysis

The amino acid sequence of Arabidopsis D14 protein (encoded by locus At3g03990) was used as a template to search D14 sequence homologs in *Populus* using the “Protein Homologs” search tool at Phytozome (www.phytozome.net). Similarly, the amino acid sequence of Arabidopsis D14L/KAI2 protein encoded by locus At4g37470 was used to search D14L/KAI2 sequence homologs in *Populus*. The percentages of amino acid similarity and identity were calculated by using MatGAT^48^. The amino acid sequence alignment of D14 and its homologs was performed by using BioEdit (http://www.mbio.ncsu.edu/bioedit/bioedit.html). Phylogenetic trees were generated by using Phylogeny.fr (http://www.phylogeny.fr/)^49^,^50^. In the phylogenetic analysis, the full length amino acid sequence of each protein was used. The Gblocks program was used to eliminate poorly aligned positions and divergent regions. Brach support values were displayed.

### Co-expression analysis

FPKM values for each gene in the *Populus* gene atlas were downloaded from Phytozome. An expression matrix was created in which each row represented genes and each column represented gene expression in different tissues or perturbations. Pearson correlations were calculated in parallel between all pairs of gene expression vectors with the use of mcxarray^51^ on the Eos cluster in the Oak Ridge Leadership Computing Facility (https://www.olcf.ornl.gov/). A threshold greater than or equal to 0.95 was applied to the resulting correlations and the remaining correlations were used to create a co-expression network. Cytoscape^52^ was used to visualize the resulting network.

### Protein structural modeling

All structural models were built using the iterative threading assembly refinement (I-TASSER, v4.3)^40^,^41^ protein structure modeling toolkit, which integrates the *de novo* modeling and template-based modeling on basis of the multiple threading alignments for protein structure constructions^53^. Three models including PtD14a and PtD14b from *P. trichocarpa* and AtD14 from *A. thaliana* were built from their respective amino acid sequences. The crystal structure of AtD14 had been previously solved using X-ray crystallography^42^; however, five amino acids at its N-terminus and one amino acid at its C-terminus residues were missing due to low electron densities. The 2D structural architectures of the models were plotted using PDBsum^54^. Structural alignment was carried out using the MultiSeq^55^ bioinformatics toolkit embedded in VMD^56^.

### Plant materials and growth conditions

Arabidopsis *D14* mutant, *Atd14-1*^31^, was kindly provided by Dr. Mark Waters at the University of Western Australia (Crawley, Australia). Arabidopsis wild-type Columbia-0 (Col-0), *Atd14-1* mutants and Arabidopsis transgenic lines were grown in an Arabidopsis growth chamber at 23 °C, approximately 125 μmol photons m^−2^ s^−1^ with 10 h/14 h photoperiod (short-day conditions).

### Cloning of *Populus D14* homologous genes

The full-length open reading frame (ORF) of each *PtD14* gene was determined according to the sequence information available at Phytozome. Gene-specific primers were designed to amplify the full-length ORF of each *PtD14* gene from cDNA library derived from RNA isolated from leaves and roots of *P. trichocarpa* plants. Subsequently, the full-length ORF of each *PtD14* gene was introduced into the pENTR™/D-TOPO® vector (Life Technologies). The cloned RT-PCR products in the pENTR vectors were validated by sequencing and then transferred into plant Gateway destination vector pGWB502Ω (2 × CaMV35SΩ)^57^ via LR reactions.

### Genetic complementation

pGWB502Ω binary vectors containing *35S:PtD14a* or *35S:PtD14b* plasmid were transformed into Arabidopsis *Atd14-1* mutants via *Agrobacterium tumefaciens* strain GV3101 mediated flower dipping transformation^58^. T1 transformants were selected using 20 μg/L hygromycin B. A minimum of 20 independent transgenic lines were selected for each transformation. Six independent transgenic lines were used for further studies. When T1 plants reached maturity, the number of primary rosette-leaf branches was counted.

### RT-PCR analysis

To examine the expression of *PtD14* genes in the Arabidopsis transgenic lines, total RNA was extracted from the rosette leaves of four-week-old plants using the Invisorb Spin Plant Mini Kit (Stratec Molecular). Two μg of total RNA were reversely transcribed to cDNA using Fermentas RevertAid Reverse Transcriptase (Thermo Scientific). *PtD14*-specific primers were used in PCR reactions. PCR amplification of Arabidopsis *ACTIN2* served as a control in the analysis of Arabidopsis transgenic lines.

### Quantitative RT-PCR

To examine the expression patterns of *PtD14* genes, total RNA was extracted from various tissues and organs of *Populus* plants using the Spectrum™ Plant Total RNA isolation kit (Sigma). Two μg of total RNA were reversely transcribed to cDNA using Fermentas RevertAid Reverse Transcriptase. Quantitative RT-PCR was performed using Maxima SYBR Green/ROX qPCR Master Mix (Thermo Scientific). Thermal cycling consisted of 50 °C for 2 min, 95 °C for 10 min, 40 cycles of 15 s at 95 °C and 60 s at 60 °C. *PtD14-*specific primers (*PtD14a*: TTAGCCGAACGCTTTTCAACA and TTCCACAGTAGCTTTGCCACC; *PtD14b*: CTAAGAGGGATACTGGGCCT and TTCCACGGTATTTTCGCCAC) were used in the quantitative RT-PCR reactions. PCR amplification of *Populus UBIQUITIN C* served as a control for normalizing the relative transcript level. All PCR reactions were done with three technical replicates.

### Yeast two-hybrid assay

The full-length open-reading frame of PtD14a, PtD14b, and PtMax2a^45^ was each cloned into pENTR vector (Life technologies, CA). For the bait construct, the pENTR vector containing PtD14a or PtD14b was transferred into the pDEST32 destination vector by LR clonase-mediated reactions (Life technologies). For the prey construct, the pENTR vector containing PtMAX2a was transferred into the pDEST22 destination vector. One hundred ng of each plasmid of bait and prey construct was added into 100 μl of MaV203 competent yeast cells (Life technologies). For negative control, 100 ng of pDEST22 and pDEST32 empty vector was co-transformed with each other or with the counterpart of pDEST plasmid DNA. Co-transformation was performed by adding 600 μl of 40% PEG/1× LiAc to yeast cell and plasmid mixture followed by incubation at 30 °C for 30 min. After incubation, 35.5 μl of DMSO was added into the cell mixture to improve transformation efficiency. Then, the yeast cells were incubated for 20 min in a 42 °C water bath. Co-transformed yeast cell was centrifuged and the pellet was diluted in 1 ml of 0.9% NaCl. A total of 100 μl of diluted yeast cells was spread on SD plate deficient of Trytophane and Leucine (SD/-Trp/-Leu). Correctly co-transformed yeast cells were cultured in 2 ml of SD/-Trp/-Leu solution overnight at 28 °C. Cultured yeast cells were diluted up to 100 times with 0.9% NaCl. Fifteen μl of diluted yeast cells were dropped on SD plate deficient of Tryptophan, Leucine, and Histidine (SD/-Trp/-Leu/-His) supplemented with 5 mM 3-Amino-1,2,4-triazole (3AT; Sigma-Aldrich, MO) or 5 mM of 3AT plus 5 μM GR24. The plates were incubated for 3 days at 28 °C. Yeast cells grown on SD plate were imaged with Canon power shot SX210 IS digital camera (Canon USA Inc., NY).

### Protein degradation assay

*Populus* mesophyll protoplasts were isolated from *P. tremula* × *alba* clone 717-1B4 leaves as described previously^46^. A total of 30 μg of plasmid expressing 10× Myc-tagged PtD14 proteins was purified with Qiagen Plasmid Midi Kit and transfected into 200 μl of protoplasts (~2 × 10^5^) using PEG-calcium mediated transfection method^59^. After 12 hr incubation at room temperature, GR24 was added into the transfected protoplast suspension to a final concentration of 5 μM and incubated for another 5 hr. Protoplasts were collected by centrifuging. Total protein was then extracted from protoplasts using 50 mM Tris-HCl (pH8.0), 100 mM NaCl, 10 mM EDTA (pH 8.0), 1% SDS, 1 mM PMSF, and protease inhibitor (Sigma). After centrifuging, the supernatant was collected and protein concentration was determined by Bradford method. Protein extracts were mixed with SDS loading buffer (60 mM Tris-HCl pH 8.0, 1% SDS, 10% glycerol, 20 mM DTT) and denatured by boiling for 8 min. To detect 10× Myc-tagged PtD14 proteins with western blotting, 1 μg of total protein was separated in 10% SDS-PAGE gel and transferred to PVDF membrane. Membranes were then probed with anti-Myc antibody (1:4000; Abgent), detected with ECL reagent (Thermo), and imaged with CCD imager (Bio-rad). In parallel, same amount of protein extracts were separated in 10% SDS-PAGE gel and stained with ProteoSilver Silver Stain Kit (Sigma). One protein band existing in all samples (~80 kDa) was selected to demonstrate equal protein loading.

## Additional Information

**How to cite this article**: Zheng, K. *et al.* Characterization of *DWARF14* Genes in *Populus.* *Sci. Rep.* **6**, 21593; doi: 10.1038/srep21593 (2016).

## Supplementary Material

Supplementary Information

## Figures and Tables

**Table 1:** Amino acid sequence similarity and identity among *Arabidopsis* and *Populus* D14 and D14L sequence homologs.

Identity (%)																												
	1	2	3	4	5	6	7	8	9	10	11	12	13	14	15	16	17	18	19	20	21	22	23	24	25	26	27	28
1. AtD14		51	21.9	22	21	21.5	22.4	15.2	41.4	17.4	43.1	24.8	50.7	17.9	17.8	22.2	79	24	19.4	50	15	17.1	23.1	77.5	18.2	20.4	16.9	17.9
2. AtD14_LIKE	75.9		21.8	19	24	19.8	20.8	16.5	39.4	18.7	39.6	22.8	80	20.7	18.5	21.6	53.1	23.7	21.6	81.1	15.3	15	23.1	52	17.9	23	20.1	19.1
3. Potri.004G039700	42.2	42		59	34	56.6	30	17.5	19.2	19.5	18.9	35.4	21.5	19.9	19.8	34.5	20.9	29.9	20.3	21.4	16.2	17.1	33.8	20.4	19.7	61.7	19.1	21.9
4. Potri.013G135100	40.9	39	76.4		36	86.3	29.8	17.7	20.1	21.9	19.7	37.8	17.9	20.1	19.4	36.5	23.1	34.8	19.9	17.6	19.8	20.2	36.2	21.4	22.4	58.5	21.4	20.1
5. Potri.013G013500	36.9	36	55.8	60		34	42.6	18.6	17.7	22.4	18	50	24	16.3	17.7	59.3	22.5	48.6	15.8	23.3	15.9	19.3	58.8	20.2	18.2	35.1	15.3	19.5
6. Potri.013G134900	41.4	40	76.4	94	59		28.2	17.9	19.7	21.5	18.9	36.3	18.2	19.8	19.7	35.3	20.4	32.6	19.8	17.9	18	19.5	35	20.4	21.9	60.4	22	20.4
7. Potri.T121700	40.1	39	47.3	45	56	45.5		15.9	17.9	19.4	21.3	34.4	20.2	19.2	15.8	74.4	21.7	44.2	19.1	21.6	15.4	16.5	68.1	20.3	19.3	29.8	17	18.6
8. Potri.003G219700	31	30	34.3	33	34	36.8	29		16.8	20	16.8	17.6	16.6	18	19.6	17.6	16.6	15	18.2	17.6	20.7	28.3	16	16.8	20.4	16.3	21.8	18.3
9. Potri.006G155500	65.5	63	40.3	39	34	38.9	36	33		17.5	46.8	22.4	40.1	18.9	16.3	16.9	41.4	18	18.3	40.1	15.9	18.2	21.3	40.3	18	19.4	18.5	20.5
10. Potri.006G219400	39.4	39	42.8	43	45	41.4	38.4	37.5	36.8		20.1	18.2	17.5	30.5	37.1	19.9	20.8	20.3	28.7	17.8	19.1	22.2	20.2	20.3	38.4	23	21.2	30.5
11. Potri.016G062700	64.1	66	39	39	33	37.9	38	32	68.7	40.4		22.8	41.4	20.7	21.1	18.8	44.6	18.1	19.2	40.7	17	17.7	19.9	44.6	21.3	21.1	17.2	21.3
12. Potri.007G018900	39.6	38	60.4	61	68	61.7	50.8	34.3	38.6	42.7	40.8		21.3	18.5	18.6	45.1	22.8	41.2	19.1	20.6	18.4	20.1	44.4	23.2	17.1	37	19.8	16.9
13. Potri.007G052000	75.2	93	40.9	40	36	38.5	35.6	31.7	65.5	36.4	66.7	37.1		19.2	17.7	21.5	52.8	21.6	19.8	92.2	14.7	16.9	20.5	51.7	20.4	22.8	17	18.9
14. Potri.007G024400	37.2	36	42	41	39	40.7	37.2	34.8	37.5	54.9	35.6	43	37.2		25.7	19.1	18.3	18.8	91.8	20.1	19.9	23	19.6	19.9	27.1	20	22.3	56.8
15. Potri.002G097400	35.5	34	42.5	40	39	39.9	31.4	35.8	37.7	58.5	39.3	40.2	34.6	52.8		17.9	19.5	18.9	25.7	16.9	17.9	21.8	17.6	18.5	58.5	18.5	21	25.8
16. Potri.002G202700	40.8	37	60.4	59	78	58.5	74.4	33.8	35.8	44.6	35.8	67.6	38.3	38.5	40.3		21.9	59.5	19.6	21.9	16.4	18.8	89.9	21.3	19.4	37	19	20.5
17. Potri.002G118900	89.1	76	40.3	42	37	40.8	38.7	32.7	65.1	40.1	67.8	37.4	75.9	38.2	37.4	40.8		22.8	20	52	15.7	15.7	23.1	91.7	19.6	19.7	20.6	20.3
18. Potri.005G081000	39.9	41	52.3	55	65	53.2	56.5	32.5	33	44.7	36.9	61.9	40.2	37.8	37.8	76	40.2		19.3	22.3	16.4	17.5	58.7	21.1	21.6	31.9	16.5	19.9
19. Potri.005G122600	38.6	38	41.8	42	39	42.4	37.7	34.8	39.9	55.4	37	43.6	39.2	95.3	55.3	39.6	38.9	37.8		20.7	19.8	23.9	19.6	18.9	26	19.6	20.6	57.3
20. Potri.005G145000	76.3	92	40.6	39	36	38.2	34.8	31.5	66.2	36.8	66.3	37.7	98.5	37.9	34	37.3	76.7	40.2	39.9		14.5	15.5	21.2	50.9	20.8	21.3	17.4	19.5
21. Potri.005G235200	29.5	31	32.7	33	31	32.4	27.3	41.3	31.9	36.1	31.9	33.7	30.5	38.1	32.7	34.4	30.5	33.7	38.6	28.7		22	17.2	15.3	19.5	17.2	20	19
22. Potri.005G121000	31.6	34	37.8	39	35	38.6	29.5	50.9	35.4	40.8	32.7	37.8	34.3	37	38.6	37.8	33.5	35.9	39.9	34.6	40.5		19.6	16.7	22	20.6	25.1	20.7
23. Potri.014G127200	40.7	40	59	58	77	57.7	71.3	33.5	35.3	44.5	37.5	66.7	38.5	39.1	40.3	95.3	40.4	76.3	39.1	37.9	33.9	37.8		22.2	19.7	36.7	16.7	20.5
24. Potri.014G016500	89.1	75	40.6	41	39	41.4	37.6	32.7	64	40.1	66.3	37.4	75.6	37.9	36.2	40.8	95.9	40.8	38.3	76.3	30	34.3	41		19.4	20.9	17.4	19
25. Potri.001G313100	36.2	37	44	43	43	43.1	35.2	38.3	37.1	59.4	40.3	41.1	37.7	54.7	78.9	44	36.2	43.2	56.3	36.5	35.1	41	41.2	36.2		18.4	23.2	28.5
26. Potri.011G048000	35.6	40	77.5	76	58	76.5	45.4	33.8	38.1	42.5	39.4	59.5	39	39.7	37.1	59.8	37.1	53.8	39.9	38.7	34.6	38.9	59.9	37.1	39.9		21.3	19.9
27. Potri.010G158800	32.6	37	38.9	38	33	38.6	28.8	40.3	36.2	41.2	34.7	38.3	35.3	41.5	42.1	37.4	34.4	35.3	40.9	35	40.3	45.3	35.6	32.3	41.8	39.8		23.6
28. Potri.004G155800	37.8	37	44.1	40	41	41.7	36.5	36.3	43.6	54.7	36.8	39.9	36.5	73.2	49.1	40.5	37.5	40.5	73.7	37.5	37.3	34.3	41	36.5	52.2	40	45.7	
**Similarity (%)**																												
