# Supplementary material for: Characterization of DWARF14 Genes in Populus
Source: Sci Rep. 2016 Feb 15;6:21593. doi: 10.1038/srep21593 (PMC4753501; doi:10.1038/srep21593)
Supplement: Supplementary Information [file srep21593-s1.pdf]

SUPPLEMENTARY INFORMATION

Characterization of *DWARF14* genes in *Populus*

Kaijie Zheng[1,2], Xiaoping Wang[1,2], Deborah A. Weighill[1,5], Hao-Bo Guo[3], Meng Xie[1], Yongil Yang[1], Jun Yang[1,4], Shucai Wang[2], Daniel A. Jacobson[1,5,6], Hong Guo[3], Wellington Muchero[1], Gerald A. Tuskan[1] and Jin-Gui Chen[1,*]

Figure S1. Bioinformatics analysis of *Arabidopsis* D14 sequence homologs Potri.016G062700 and Potri.006G155500 in *Populus*. (**a**) Amino acid sequence alignment of Arabidopsis D14 with its PtD14a, PtD14b, Potri.016G062700 and Potri.006G155500. The catalytic triad Ser96-His246-Asp217 of PtD14a/b proteins is indicated by arrow heads on top of the amino acids. (**b**) Percentage of amino acid similarity and identity.

Figure S2. Phylogenetic analysis of *Populus* sequence homologues of *Arabidopsis* D14 protein.

Figure S3. Phylogenetic analysis of *Populus* sequence homologues of *Arabidopsis* D14-LIKE protein.

Figure S4. RNAseq analysis of expression patterns of *PtD14a* and *PtD14b* across various tissues and organs. Expression data (in FPKM) were extracted from *Populus* gene atlas in Phytozome.

Figure S5. Structural multi-sequence alignment of PtD14 proteins with AtD14. Residues in grey (M1-S3 in PtD14) show highest deviation in all three models (Figure 5A), those in red are deviated between PtD14a and PtD14b and those in green are deviated between AtD14 and PtD14a. For the residues deviated between PtD14a and PtD14b (in red), only F27/V27 (arrow) are close to the active site (the catalytic triad) as shown in Figure 5C-E. Other residues are located at the protein periphery and are far away from the catalytic triad.

Table S1. List of genes co-expressed with *PtD14a, PtD14b* or both. Pearson correlation coefficient ≥0.95 was used to select co-expressed genes.

Figure S1

a

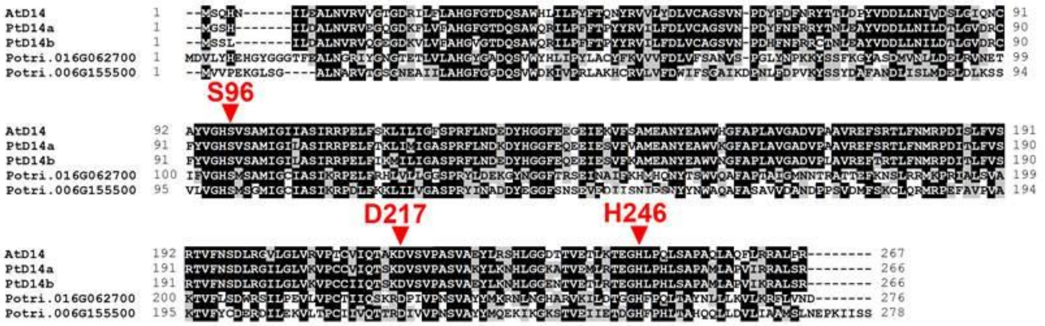

b

	AtD14	PtD14a	PtD14b	Potri.016G062700	Potri.006G155500
			Identity (%)		
**AtD14**		79.0	77.5	43.1	41.4
**PtD14a**	89.1		91.7	67.8	65.1
**PtD14b**	89.1	95.9		66.3	64.0
**Potri.016G062700**	64.1	44.6	44.6		68.7
**Potri.006G155500**	65.5	41.4	40.3	46.8	
			Similarity (%)		

Figure S2

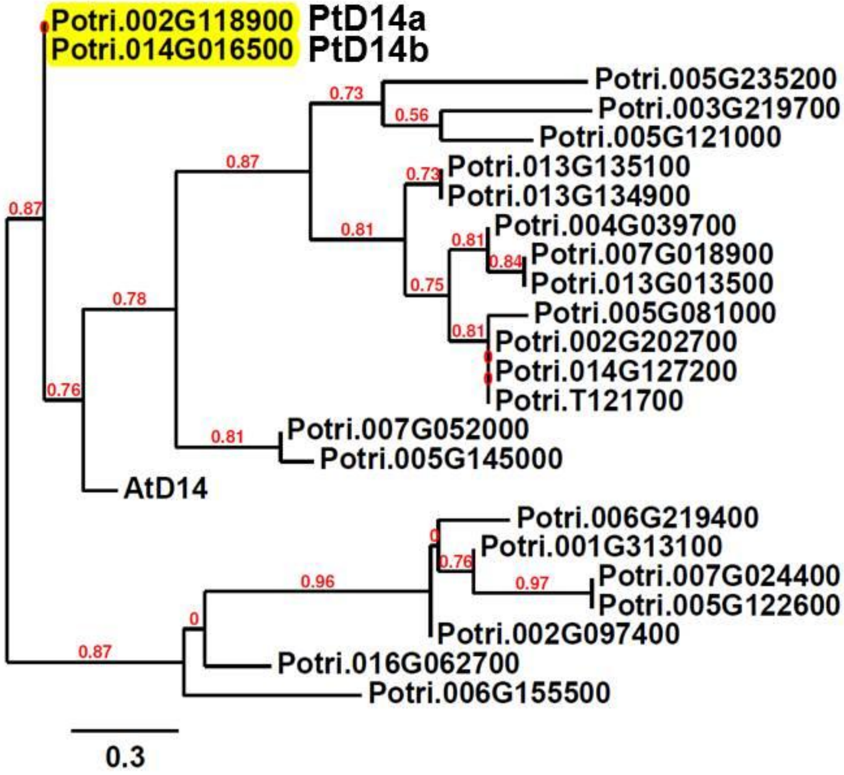

Potri.002G118900 PtD14a
Potri.014G016500 PtD14b
Potri.005G235200
Potri.003G219700
Potri.005G121000
Potri.013G135100
Potri.013G134900
Potri.004G039700
Potri.007G018900
Potri.013G013500
Potri.005G081000
Potri.002G202700
Potri.014G127200
Potri.T121700
Potri.007G052000
Potri.005G145000
AtD14
Potri.006G219400
Potri.001G313100
Potri.007G024400
Potri.005G122600
Potri.002G097400
Potri.016G062700
Potri.006G155500
0.73
0.56
0.87
0.73
0.81
0.81
0.84
0.75
0.81
0.87
0.78
0.76
0.81
0
0.76
0.97
0.96
0
0.87
0.3

Figure S3

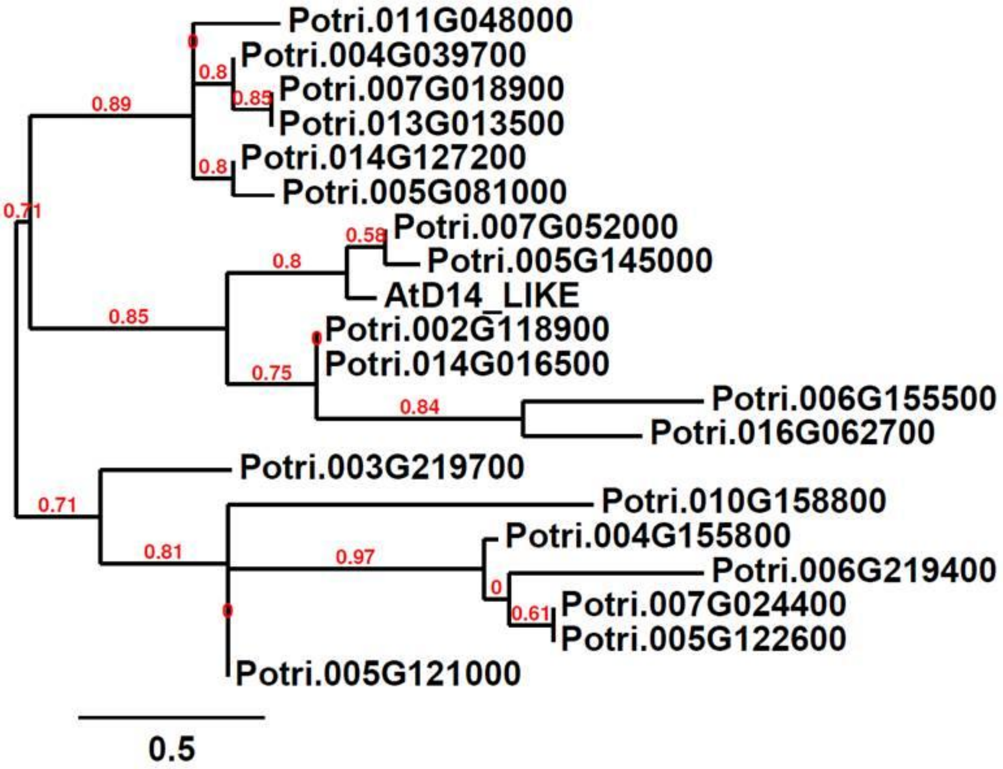

Potri.011G048000
Potri.004G039700
Potri.007G018900
Potri.013G013500
Potri.014G127200
Potri.005G081000
Potri.007G052000
Potri.005G145000
AtD14_LIKE
Potri.002G118900
Potri.014G016500
Potri.006G155500
Potri.016G062700
Potri.003G219700
Potri.010G158800
Potri.004G155800
Potri.006G219400
Potri.007G024400
Potri.005G122600
Potri.005G121000
0.89
0.8
0.85
0.71
0.58
0.75
0.84
0.81
0.97
0.61
0
0.5

Figure S4

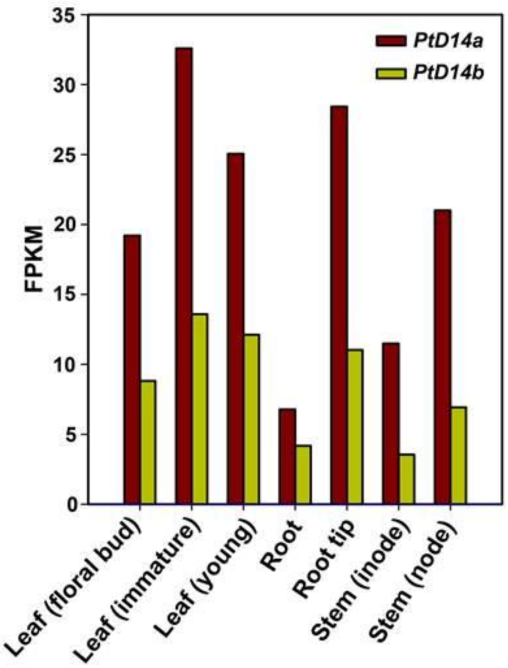

35
30
25
20
15
10
5
0
FPKM
PtD14a
PtD14b
Leaf (floral bud)
Leaf (immature)
Leaf (young)
Root
Root tip
Stem (inode)
Stem (node)

Figure S5

```
AtD14     MSQ-HNILEALNVRVVGTGDRILFLAHGFGTDQSAWHLILPYFTQNYRVVLYDLVCAGSV
PtD14a    --MGSHILDALNVRVEGQGDKFLVFAHGFGTDQSAWQRILPFFTPYYRVILFDLVCAGSV
PtD14b    -MS-SLILDALNVRVQGEGDKVLVFAHGVGTDQSAWQRILPFFTPYYRVILFDLVCAGSV

AtD14     NPDYFDFNRYTTLDPYVDDLLNIVDSLGIQNCAYVGHSVSAMIGIIASIRRPELFSKLIL
PtD14a    NPDYFNFRRYTNLEAYVDDLLNILDTLGVDRCFYVGHSVSAMIGILASIRRPELFTKLIM
PtD14b    NPDHFNFRRCTNLEAYVDDLLNILDTLGVDRCFYVGHSVSAMIGILASIRRPELFIKMIL

AtD14     IGFSPRFLNDEDYHGGFEEGEIEKVFSAMEANYEAWVHGFAPLAVGADVPAAVREFSRTL
PtD14a    IGASPRFLNDKDYHGGFEQEEIESVFVAMEANYEAWVKGFAPLAVGADVPAAVREFSRTL
PtD14b    IGASPRFLNDEDYHGGFEQEEIESVFKAMEANYEAWVNGFAPLAVGADVPLAVREFTRTL

AtD14     FNMRPDISLFVSRTVFNSDLRGVLGLVRVPTCVIQTAKDVSVPASVAEYLRSHLGGDTTV
PtD14a    FNMRPDITLFVSRTVFNSDLRGILGLVKVPCCVIQTSKDVSVPASVAKYLKNHLGGKATV
PtD14b    FNMRPDITLFVSRTVFNSDLRGILGLVKVPCCIIQTSKDVSVPASVAEYLKNHLGGENTV

AtD14     ETLKTEGHLPQLSAPAQLAQFLRRALPR
PtD14a    EMLRTEGHLPHLSAPAMLAPVIRRALSR
PtD14b    ETLRTEGHLPHLSAPAMLAPVIKRALSR
```

Table S1. List of genes co-expressed with *PtD14a, PtD14b* or both. Pearson correlation coefficient ≥0.95 was used to select co-expressed genes.

**Genes co-expressed with Potri.002G118900 (*PtD14a*) only**	
**GeneID**	**Mapman_name**
Potri.016G031500	lipid metabolism.lipid degradation.lysophospholipases.carboxylesterase
Potri.T020000	not assigned.unknown
Potri.010G120200	not assigned.unknown
Potri.009G021800	secondary metabolism.isoprenoids.non-mevalonate pathway.CMK
Potri.006G267400	misc.peroxidases
Potri.013G017900	not assigned.no ontology.formin homology 2 domain-containing protein
Potri.001G392700	protein.glycosylation
Potri.016G017800	not assigned.no ontology.pentatricopeptide (PPR) repeat-containing protein
Potri.010G145300	not assigned.unknown
Potri.001G053800	misc.UDP glucosyl and glucoronyl transferases
Potri.009G092400	not assigned.unknown
Potri.017G053800	DNA.synthesis/chromatin structure
Potri.002G022900	cell.vesicle transport
Potri.009G139600	secondary metabolism.isoprenoids.non-mevalonate pathway.geranylgeranyl pyrophosphate synthase
Potri.009G154300	signalling.receptor kinases.wall associated kinase
Potri.015G000500	not assigned.unknown
Potri.019G030400	not assigned.unknown
Potri.014G040700	stress.biotic.PR-proteins : stress.biotic
Potri.017G062100	not assigned.no ontology
Potri.001G160300	signalling.receptor kinases.C-Lectin
Potri.009G103800	lipid metabolism.lipid degradation.lysophospholipases.carboxylesterase : Biodegradation of Xenobiotics : lipid metabolism.lipid degradation.beta-oxidation.acyl-CoA thioesterase
Potri.004G134800	misc.peroxidases
Potri.T076700	misc.oxidases - copper, flavone etc
Potri.006G200300	cell wall.cell wall proteins.AGPs.AGP
Potri.010G175000	protein.degradation
Potri.001G006100	misc.myrosinases-lectin-jacalin
Potri.016G002400	protein.synthesis.ribosomal protein.prokaryotic.unknown organellar.50S subunit.L15
Potri.006G025800	hormone metabolism.abscisic acid.induced-regulated-responsive-activated : RNA.regulation of transcription.bZIP transcription factor

	family
Potri.003G081800	signalling.G-proteins
Potri.007G045900	not assigned.unknown
Potri.011G049600	signalling.receptor kinases.leucine rich repeat VIII.VIII-2
Potri.008G021400	not assigned.no ontology
Potri.018G024000	not assigned.unknown
Potri.004G127400	not assigned.no ontology.glycine rich proteins
Potri.009G168500	transport.sugars
Potri.005G165200	transport.metabolite transporters at the mitochondrial membrane
Potri.008G205200	misc.cytochrome P450
Potri.014G048300	not assigned.unknown
Potri.007G079200	not assigned.no ontology
Potri.001G160200	not assigned.unknown
Potri.004G037100	not assigned.unknown
Potri.001G342100	not assigned.unknown
Potri.008G007000	RNA.regulation of transcription.C2C2(Zn) CO-like, Constans-like zinc finger family
Potri.018G139500	transport.sugars
Potri.007G001500	development.unspecified
Potri.006G069300	signalling.G-proteins
Potri.017G034700	signalling.receptor kinases.Catharanthus roseus-like RLK1 : lipid metabolism.lipid degradation.lysophospholipases.glycerophosphodiester phosphodiesterase : signalling.receptor kinases.thaumatin like : signalling.receptor kinases.wheat LRK10 like
Potri.001G329300	RNA.regulation of transcription.C2H2 zinc finger family
Potri.019G059400	misc.rhodanese
Potri.005G117600	RNA.regulation of transcription.C2C2(Zn) GATA transcription factor family
Potri.008G073800	secondary metabolism.simple phenols
Potri.002G047500	signalling.calcium
Potri.004G113200	hormone metabolism.auxin.induced-regulated-responsive-activated
Potri.006G111900	not assigned.unknown
Potri.017G034500	signalling.receptor kinases.wheat LRK10 like : signalling.receptor kinases.thaumatin like : signalling.receptor kinases.Catharanthus roseus-like RLK1 : lipid metabolism.lipid degradation.lysophospholipases.glycerophosphodiester phosphodiesterase
Potri.001G021000	signalling.calcium
Potri.019G042700	not assigned.unknown
Potri.002G106900	transport.sugars.sucrose
Potri.015G061800	misc.UDP glucosyl and glucoronyl transferases

Potri.012G104100	protein.degradation.ubiquitin
Potri.009G126200	RNA.regulation of transcription.General Transcription, TBP-binding protein
Potri.003G072800	cell wall.pectin*esterases.PME
Potri.001G011000	misc.peroxidases
Potri.010G063500	transport.ammonium
Potri.004G173500	cell.cycle.peptidylprolyl isomerase
Potri.003G164400	RNA.processing
Potri.005G145300	protein.degradation.subtilases
Potri.001G017500	signalling.receptor kinases.leucine rich repeat XI
Potri.008G034100	not assigned.no ontology
Potri.013G158500	RNA.regulation of transcription.AP2/EREBP, APETALA2/Ethylene-responsive element binding protein family
Potri.004G030600	signalling.light
Potri.009G046900	not assigned.unknown
Potri.005G004800	not assigned.unknown
Potri.004G228200	protein.aa activation.pseudouridylate synthase
Potri.018G146400	not assigned.unknown
Potri.005G212500	not assigned.unknown
Potri.003G024600	misc.dynamin
Potri.016G122200	not assigned.no ontology
Potri.018G128700	not assigned.unknown
Potri.001G391100	misc.oxidases - copper, flavone etc
Potri.012G077000	signalling.calcium
Potri.005G212600	not assigned.unknown
Potri.006G221400	RNA.regulation of transcription.Trihelix, Triple-Helix transcription factor family
Potri.002G103600	not assigned.no ontology.pentatricopeptide (PPR) repeat-containing protein
Potri.003G165300	not assigned.unknown
Potri.013G124000	protein.postranslational modification
Potri.012G058900	transport.unspecified cations
Potri.019G094100	stress.biotic
Potri.008G208000	misc.UDP glucosyl and glucoronyl transferases
Potri.015G043100	misc.oxygenases
Potri.016G043200	transport.metabolite transporters at the envelope membrane
Potri.013G056000	stress.biotic
Potri.006G085900	RNA.regulation of transcription.MYB domain transcription factor family
Potri.018G054700	RNA.regulation of transcription.HB,Homeobox transcription factor family

Potri.018G111000	protein.degradation
Potri.010G228400	protein.degradation.cysteine protease
Potri.001G101500	RNA.RNA binding
Potri.T019800	not assigned.unknown
Potri.003G055500	signalling.calcium : development.unspecified
Potri.001G244900	misc.nitrilases, *nitrile lyases, berberine bridge enzymes, reticuline oxidases, troponine reductases
Potri.003G038800	development.unspecified
Potri.006G211800	not assigned.unknown
Potri.002G019500	signalling.G-proteins
Potri.001G072500	misc.UDP glucosyl and glucoronyl transferases
Potri.006G151800	development.unspecified
Potri.015G033000	RNA.regulation of transcription.BBR/BPC
Potri.005G034800	protein.degradation.ubiquitin.E3.RING
Potri.011G002500	transport.misc
Potri.017G002300	misc.oxidases - copper, flavone etc
Potri.001G313900	not assigned.unknown
Potri.002G238900	not assigned.unknown
Potri.008G021600	not assigned.unknown
Potri.003G052400	development.unspecified
Potri.006G067700	RNA.regulation of transcription.Methyl binding domain proteins
Potri.008G115900	RNA.regulation of transcription.unclassified
Potri.008G161200	RNA.regulation of transcription.Aux/IAA family
Potri.004G207600	protein.degradation.cysteine protease
Potri.002G174300	RNA.regulation of transcription.C2C2(Zn) DOF zinc finger family
Potri.004G156400	secondary metabolism.simple phenols
Potri.017G040800	major CHO metabolism.degradation.starch.starch cleavage.beta amylase
Potri.011G111900	not assigned.no ontology
Potri.008G087500	not assigned.unknown
Potri.002G115000	RNA.RNA binding
Potri.006G166700	misc.GDSL-motif lipase
Potri.014G146600	stress.biotic
Potri.008G115800	RNA.regulation of transcription.TCP transcription factor family
Potri.011G122300	hormone metabolism.abscisic acid.signal transduction
Potri.015G073200	development.unspecified
Potri.004G059100	signalling.receptor kinases.leucine rich repeat II
Potri.010G161100	protein.degradation.ubiquitin.E3.RING
Potri.009G027600	RNA.regulation of transcription.Methyl binding domain proteins
Potri.013G149200	signalling.receptor kinases.misc : RNA.regulation of

	transcription.MYB domain transcription factor family : secondary metabolism.sulfur-containing.glucosinolates.regulation.indole
Potri.016G135100	cell.cell death.plants
Potri.001G449800	not assigned.unknown
Potri.009G086700	cell.cycle
Potri.001G041700	protein.degradation.aspartate protease
Potri.018G038400	not assigned.unknown
Potri.001G460700	misc.short chain dehydrogenase/reductase (SDR)
Potri.003G209700	hormone metabolism.ethylene.signal transduction
Potri.003G202900	hormone metabolism.abscisic acid.signal transduction
Potri.001G006600	lipid metabolism.FA synthesis and FA elongation.acyl coa ligase
Potri.002G168400	not assigned.unknown
Potri.001G185900	hormone metabolism.ethylene.induced-regulated-responsive-activated
Potri.003G053000	not assigned.unknown
Potri.018G065800	not assigned.unknown
Potri.007G024100	RNA.regulation of transcription.zf-HD
Potri.008G029300	major CHO metabolism.degradation.starch.laforin like phosphoglucan phosphatase (SEX4)
Potri.008G138400	cell wall.modification
Potri.004G142800	lipid metabolism.lipid degradation.lysophospholipases.carboxylesterase : Biodegradation of Xenobiotics : lipid metabolism.lipid degradation.beta-oxidation.acyl-CoA thioesterase
Potri.017G093500	not assigned.no ontology
Potri.010G050900	not assigned.no ontology
Potri.003G096900	not assigned.unknown
Potri.018G131300	signalling.G-proteins
Potri.006G251300	signalling.G-proteins
Potri.013G077500	development.unspecified : RNA.regulation of transcription.General Transcription
Potri.001G296400	protein.targeting.peroxisomes
Potri.019G092000	not assigned.unknown
Potri.015G024700	stress.biotic.PR-proteins
Potri.002G207000	RNA.regulation of transcription.ARF, Auxin Response Factor family

**Genes co-expressed with Potri.014G016500 (*PtD14b*) only**	
**GeneID**	**Mapman_name**
Potri.008G200000	transport.misc
Potri.002G130700	hormone metabolism.jasmonate.synthesis-degradation.allene oxidase synthase
Potri.008G078800	amino acid metabolism.synthesis.branched chain group.common.acetolactate synthase
Potri.005G068800	stress.abiotic.light
Potri.012G135900	RNA.regulation of transcription.TCP transcription factor family
Potri.015G143300	RNA.processing.3 end processing.CFIm25
Potri.016G064600	protein.degradation.ubiquitin.E3.RING
Potri.012G080400	RNA.regulation of transcription.MYB domain transcription factor family
Potri.016G045400	stress.abiotic.heat
Potri.005G176900	not assigned.unknown
Potri.004G088500	stress.biotic.PR-proteins
Potri.T137700	not assigned.no ontology.BSD domain-containing protein
Potri.005G231300	RNA.regulation of transcription.bZIP transcription factor family
Potri.016G138100	protein.synthesis.elongation
Potri.002G015100	misc.glutathione S transferases
Potri.001G140400	not assigned.unknown
Potri.015G042100	minor CHO metabolism.others
Potri.016G115300	protein.degradation.ubiquitin.E3.RING
Potri.014G038500	not assigned.unknown
Potri.003G082500	cell.vesicle transport
Potri.008G020500	not assigned.unknown
Potri.005G095800	amino acid metabolism.synthesis.glutamate family.arginine.acetylornithine aminotransferase
Potri.015G075100	not assigned.no ontology
Potri.002G217300	not assigned.unknown
Potri.008G200300	transport.misc
Potri.017G075600	misc.UDP glucosyl and glucoronyl transferases
Potri.013G054900	stress.abiotic.heat
Potri.011G045700	not assigned.unknown
Potri.003G093300	protein.degradation.ubiquitin.E3.RING : stress.biotic
Potri.001G377100	not assigned.unknown
Potri.008G062500	not assigned.unknown
Potri.002G161700	not assigned.unknown
Potri.014G165700	protein.degradation.ubiquitin.E3.SCF.FBOX
Potri.003G151700	cell wall.cellulose synthesis

Potri.017G101700	RNA.regulation of transcription.bHLH,Basic Helix-Loop-Helix family
Potri.007G030500	misc.UDP glucosyl and glucoronyl transferases : secondary metabolism.flavonoids.dihydroflavonols : secondary metabolism.phenylpropanoids.lignin biosynthesis
Potri.014G113900	RNA.regulation of transcription.MYB-related transcription factor family
Potri.001G455800	protein.degradation.subtilases
Potri.008G034500	misc.UDP glucosyl and glucoronyl transferases
Potri.019G013100	not assigned.no ontology.glycine rich proteins
Potri.014G066700	RNA.regulation of transcription.C2C2(Zn) YABBY family
Potri.016G072900	RNA.regulation of transcription.ovate family OFP
Potri.012G037200	major CHO metabolism.degradation.sucrose.Susy
Potri.006G008000	not assigned.unknown
Potri.011G045900	not assigned.unknown
Potri.011G122900	cell.organisation
Potri.003G016800	not assigned.no ontology.pentatricopeptide (PPR) repeat-containing protein
Potri.014G124400	RNA.regulation of transcription.C2C2(Zn) GATA transcription factor family
Potri.012G140600	cell.vesicle transport
Potri.001G124700	not assigned.unknown
Potri.005G088300	hormone metabolism.abscisic acid.induced-regulated-responsive-activated
Potri.014G156900	not assigned.unknown
Potri.016G004100	secondary metabolism.isoprenoids.mevalonate pathway.farnesyl pyrophosphate synthetase
Potri.009G049900	stress.abiotic.heat
Potri.008G128400	not assigned.no ontology
Potri.004G098100	protein.synthesis.ribosome biogenesis
Potri.002G041000	lipid metabolism.lipid degradation.lipases.triacylglycerol lipase
Potri.001G389200	misc.UDP glucosyl and glucoronyl transferases : hormone metabolism.salicylic acid.synthesis-degradation
Potri.004G003300	not assigned.unknown
Potri.011G060400	stress.biotic.PR-proteins : stress.biotic.receptors
Potri.008G058800	not assigned.no ontology
Potri.003G099700	secondary metabolism.phenylpropanoids.lignin biosynthesis.4CL
Potri.001G155400	not assigned.no ontology
Potri.002G246500	cell wall.pectin synthesis
Potri.001G111800	RNA.regulation of transcription.TCP transcription factor family
Potri.005G025700	not assigned.no ontology
Potri.015G044300	not assigned.no ontology
Potri.013G005800	minor CHO metabolism.raffinose family.galactinol synthases.known

Potri.002G008300	signalling.calcium
Potri.016G116300	RNA.regulation of transcription.C3H zinc finger family : protein.degradation.ubiquitin.E3.RING
Potri.004G185900	signalling.phosphorelay : signalling.receptor kinases.misc
Potri.005G078600	development.unspecified
Potri.005G112200	redox.ascorbate and glutathione.ascorbate
Potri.010G235500	misc.UDP glucosyl and glucoronyl transferases
Potri.004G180900	not assigned.unknown
Potri.002G115900	RNA.regulation of transcription.bZIP transcription factor family
Potri.002G207800	hormone metabolism.auxin.signal transduction
Potri.009G061200	not assigned.unknown
Potri.008G145000	not assigned.unknown
Potri.002G060800	development.unspecified
Potri.006G138600	protein.folding
Potri.014G094100	misc.acyl transferases
Potri.016G071800	stress.abiotic.heat
Potri.001G135500	secondary metabolism.isoprenoids.mevalonate pathway.phosphomevalonate kinase
Potri.007G089100	not assigned.unknown
Potri.004G041700	not assigned.unknown
Potri.001G423800	not assigned.unknown
Potri.002G070000	protein.postranslational modification
Potri.014G100800	RNA.regulation of transcription.MYB domain transcription factor family : secondary metabolism.sulfur-containing.glucosinolates.regulation.indole : signalling.receptor kinases.misc
Potri.012G145600	amino acid metabolism.synthesis.aromatic aa.tryptophan.phosphoribosyanthranilate isomerase
Potri.009G099800	secondary metabolism.phenylpropanoids.lignin biosynthesis.CCoAOMT
Potri.017G134900	signalling.receptor kinases.leucine rich repeat IX
Potri.010G218100	signalling.calcium
Potri.005G149500	development.unspecified
Potri.019G118600	cell.cycle
Potri.003G159400	not assigned.unknown
Potri.019G099100	not assigned.no ontology
Potri.008G063400	transport.metabolite transporters at the envelope membrane
Potri.007G012700	RNA.regulation of transcription.General Transcription, TBP-binding protein
Potri.019G118800	signalling.receptor kinases.misc : secondary metabolism.sulfur-containing.glucosinolates.regulation.aliphatic : RNA.regulation of transcription.MYB domain transcription factor family : secondary

	metabolism.sulfur-containing.glucosinolates.regulation.indole
Potri.006G226400	signalling.calcium
Potri.005G133600	misc.nitrilases, *nitrile lyases, berberine bridge enzymes, reticuline oxidases, troponine reductases : hormone metabolism
Potri.014G087200	protein.postranslational modification.kinase.receptor like cytoplasmatic kinase IV
Potri.014G090500	Co-factor and vitamine metabolism.pantothenate.3-methyl-2-oxobutanoate hydroxymethyltransferase (KPHMT,PANB)
Potri.003G212700	lipid metabolism.FA synthesis and FA elongation.enoyl ACP reductase
Potri.007G145900	amino acid metabolism.synthesis.aspartate family.lysine.dihydrodipicolinate reductase
Potri.015G128600	protein.degradation.ubiquitin.E3.RING
Potri.012G054100	not assigned.no ontology
Potri.001G309000	not assigned.unknown
Potri.001G422500	misc.cytochrome P450
Potri.002G098000	stress.abiotic.drought/salt
Potri.016G009200	not assigned.unknown
Potri.001G232000	lipid metabolism.lipid transfer proteins etc : misc.protease inhibitor/seed storage/lipid transfer protein (LTP) family protein
Potri.014G076900	not assigned.unknown
Potri.007G097700	not assigned.unknown
Potri.014G089100	protein.synthesis.ribosomal protein.prokaryotic.chloroplast.50S subunit.L5
Potri.002G064300	hormone metabolism.auxin.induced-regulated-responsive-activated
Potri.005G158800	RNA.regulation of transcription.zf-HD
Potri.017G139100	major CHO metabolism.degradation.sucrose.Susy
Potri.016G080500	cell wall.precursor synthesis.UXS
Potri.017G098200	signalling.receptor kinases.Catharanthus roseus-like RLK1
Potri.003G101600	protein.postranslational modification
Potri.007G146000	Co-factor and vitamine metabolism.riboflavin.riboflavin synthase
Potri.013G108900	RNA.regulation of transcription.zf-HD
Potri.001G210500	not assigned.unknown
Potri.014G044300	protein.folding
Potri.006G194500	not assigned.unknown
Potri.007G020800	protein.degradation.AAA type
Potri.013G108300	transport.p- and v-ATPases
Potri.005G226600	transport.ABC transporters and multidrug resistance systems
Potri.005G232000	RNA.regulation of transcription.HB,Homeobox transcription factor family
Potri.003G026500	not assigned.unknown
Potri.006G034100	not assigned.no ontology
Potri.002G262500	development.unspecified : RNA.regulation of transcription.ovate

	family OFP
Potri.001G178800	tetrapyrrole synthesis.sirohydrochlorin ferrochelatase
Potri.011G049500	not assigned.unknown
Potri.001G409600	not assigned.unknown
Potri.006G084400	not assigned.unknown
Potri.008G064500	not assigned.unknown
Potri.001G379400	not assigned.unknown
Potri.009G055900	protein.degradation.serine protease
Potri.018G101800	not assigned.unknown
Potri.010G160500	misc.acid and other phosphatases
Potri.005G081400	not assigned.unknown
Potri.T148700	hormone metabolism.cytokinin.synthesis-degradation : misc.UDP glucosyl and glucoronyl transferases
Potri.011G129800	stress.biotic.PR-proteins
Potri.006G216700	misc.beta 1,3 glucan hydrolases : misc.gluco-, galacto- and mannosidases
Potri.009G167900	transport.amino acids
Potri.002G008600	signalling.calcium
Potri.002G229500	protein.synthesis.ribosomal protein.prokaryotic.chloroplast.50S subunit.L6
Potri.013G052600	not assigned.unknown
Potri.008G093300	RNA.regulation of transcription.unclassified
Potri.002G004600	RNA.RNA binding
Potri.013G042900	not assigned.unknown
Potri.010G107900	development.unspecified
Potri.002G167200	minor CHO metabolism.others
Potri.001G044400	DNA.repair
Potri.013G145200	not assigned.unknown
Potri.008G132400	DNA.synthesis/chromatin structure
Potri.003G051700	Co-factor and vitamine metabolism.isochorismatase
Potri.018G048100	not assigned.unknown
Potri.001G410000	RNA.processing
Potri.019G019800	not assigned.unknown
Potri.007G107500	not assigned.unknown
Potri.016G129600	signalling.receptor kinases.misc
Potri.010G072700	protein.degradation.ubiquitin.E3.RING
Potri.019G031000	nucleotide metabolism.degradation
Potri.017G060300	misc.beta 1,3 glucan hydrolases.glucan endo-1,3-beta-glucosidase
Potri.013G001200	not assigned.unknown
Potri.016G049300	not assigned.no ontology.C2 domain-containing protein

Potri.001G454900	not assigned.unknown
Potri.006G120000	misc.O-methyl transferases
Potri.018G075400	not assigned.unknown
Potri.016G136400	protein.postranslational modification
Potri.018G092600	not assigned.unknown
Potri.007G069700	transport.Major Intrinsic Proteins.TIP
Potri.005G177100	stress.abiotic.unspecified
Potri.002G061100	amino acid metabolism.synthesis.aromatic aa.chorismate.shikimate kinase
Potri.003G186500	not assigned.no ontology
Potri.001G213700	development.unspecified
Potri.007G058700	protein.postranslational modification
Potri.009G157000	redox.misc
Potri.012G108700	not assigned.unknown
Potri.001G423900	not assigned.no ontology
Potri.005G226000	stress.abiotic.drought/salt
Potri.019G043900	protein.degradation.ubiquitin.E3.RING
Potri.009G092200	not assigned.no ontology
Potri.006G238000	signalling.MAP kinases
Potri.011G042700	not assigned.unknown
Potri.006G216300	not assigned.unknown
Potri.005G011200	not assigned.unknown
Potri.001G236700	cell.organisation : cell.organisation.cytoskeleton.actin.actin depolymerizing factors
Potri.001G028100	not assigned.unknown
Potri.005G003600	not assigned.unknown
Potri.005G089600	redox.dismutases and catalases
Potri.005G024700	protein.degradation.ubiquitin.E3.RING
Potri.005G255300	not assigned.unknown
Potri.014G038800	not assigned.unknown
Potri.015G041300	misc.gluco-, galacto- and mannosidases
Potri.003G152100	not assigned.unknown
Potri.001G117000	not assigned.unknown
Potri.006G241000	cell.vesicle transport
Potri.017G107600	protein.degradation.ubiquitin.E3.SCF.FBOX
Potri.012G022600	RNA.regulation of transcription.General Transcription
Potri.T106100	not assigned.no ontology
Potri.013G077000	secondary metabolism.isoprenoids.tocopherol biosynthesis.tocopherol methyltransferase
Potri.001G406300	RNA.regulation of transcription.unclassified

Potri.017G143800	stress.biotic.PR-proteins : stress.biotic
Potri.013G069400	transport.misc
Potri.017G119100	not assigned.no ontology
Potri.015G039200	stress.biotic
Potri.011G022800	protein.postranslational modification
Potri.003G208900	not assigned.unknown
Potri.018G015100	RNA.regulation of transcription.unclassified
Potri.007G013300	DNA.synthesis/chromatin structure.histone.core.H4
Potri.011G117300	transport.misc
Potri.005G215900	secondary metabolism.isoprenoids.tocopherol biosynthesis.MPBQ/MSBQ methyltransferase
Potri.015G006200	RNA.regulation of transcription.General Transcription
Potri.014G137700	not assigned.no ontology
Potri.005G156400	not assigned.unknown
Potri.003G078000	PS.photorespiration
Potri.017G026200	not assigned.unknown
Potri.T034300	not assigned.unknown
Potri.018G050000	not assigned.unknown
Potri.001G338900	not assigned.unknown
Potri.011G122400	tetrapyrrole synthesis.protochlorophyllide reductase
Potri.008G117500	RNA.regulation of transcription.G2-like transcription factor family, GARP
Potri.006G275900	RNA.regulation of transcription.MYB domain transcription factor family
Potri.014G179800	protein.folding
Potri.010G113900	protein.degradation.ubiquitin : protein.degradation.ubiquitin.E3.RING
Potri.017G052000	hormone metabolism.cytokinin.synthesis-degradation : misc.UDP glucosyl and glucoronyl transferases
Potri.018G106700	not assigned.unknown
Potri.001G365400	secondary metabolism.flavonoids.dihydroflavonols.flavonoid 3-monooxygenase : misc.cytochrome P450
Potri.002G031800	protein.targeting.chloroplast
Potri.014G149500	not assigned.no ontology.pentatricopeptide (PPR) repeat-containing protein
Potri.005G201200	cell wall.modification
Potri.014G035400	not assigned.unknown
Potri.001G033900	RNA.processing.ribonucleases
Potri.004G135100	RNA.regulation of transcription.zf-HD
Potri.018G098500	protein.degradation.ubiquitin.E3.RING
Potri.010G068900	not assigned.no ontology
Potri.014G120000	RNA.regulation of transcription.GRAS transcription factor family

Potri.015G029400	transport.sugars
Potri.003G197000	not assigned.unknown
Potri.014G020900	protein.degradation.ubiquitin.E3.RING : RNA.regulation of transcription.C3H zinc finger family
Potri.009G003800	not assigned.no ontology
Potri.008G220700	not assigned.unknown
Potri.006G273200	stress.biotic
Potri.006G026500	protein.degradation
Potri.007G051600	not assigned.unknown

**Genes co-expressed with both Potri.002G118900 (*PtD14a*) and Potri.014G016500 (*PtD14b*)**	
**GeneID**	**Mapman_name**
Potri.001G310100	not assigned.unknown
Potri.006G097500	secondary metabolism.phenylpropanoids : secondary metabolism.flavonoids.anthocyanins
Potri.003G095300	cell.vesicle transport
Potri.006G223500	RNA.regulation of transcription.Alfin-like
Potri.005G001700	not assigned.unknown
Potri.019G052600	not assigned.no ontology
Potri.001G462600	secondary metabolism.N misc.alkaloid-like : misc.nitrilases, *nitrile lyases, berberine bridge enzymes, reticuline oxidases, troponine reductases
Potri.009G117000	not assigned.no ontology
Potri.009G099000	secondary metabolism.flavonoids.flavonols.flavonol 3-O-glycosyltransferase : misc.UDP glucosyl and glucoronyl transferases
Potri.010G041700	not assigned.unknown
Potri.016G141300	not assigned.unknown
Potri.002G179200	not assigned.unknown
Potri.011G055900	development.squamosa promoter binding like (SPL)
Potri.012G125800	not assigned.unknown
Potri.008G029600	not assigned.unknown
Potri.002G035200	RNA.regulation of transcription.zf-HD
Potri.004G121100	misc.plastocyanin-like
Potri.007G005000	RNA.regulation of transcription.MYB-related transcription factor family
Potri.017G131000	nucleotide metabolism.salvage.NUDIX hydrolases
Potri.016G024500	protein.degradation
Potri.015G143700	redox.ascorbate and glutathione
Potri.005G147500	misc.UDP glucosyl and glucoronyl transferases
Potri.013G103800	secondary metabolism.flavonoids.isoflavones.isoflavone reductase
Potri.T096600	misc.cytochrome P450
Potri.002G149100	not assigned.unknown
Potri.015G032700	RNA.regulation of transcription.zf-HD
Potri.004G106400	amino acid metabolism.degradation.glutamate family.proline
Potri.001G166800	stress.biotic.PR-proteins.proteinase inhibitors.trypsin inhibitor
Potri.002G169200	not assigned.no ontology
Potri.004G106600	misc.cytochrome P450
Potri.014G057700	development.squamosa promoter binding like (SPL)
Potri.002G183600	secondary metabolism.phenylpropanoids.lignin

	biosynthesis.CCoAOMT
Potri.005G227900	RNA.regulation of transcription.zf-HD
Potri.012G072700	RNA.regulation of transcription.bHLH,Basic Helix-Loop-Helix family
Potri.001G245200	protein.postranslational modification
Potri.007G044700	signalling.misc
Potri.008G191900	protein.assembly and cofactor ligation
Potri.010G102200	amino acid metabolism.synthesis.aromatic aa.tryptophan.anthranilate synthase
Potri.018G059100	not assigned.unknown
Potri.001G114000	RNA.regulation of transcription.GRF zinc finger family : RNA.regulation of transcription.General Transcription
Potri.004G120000	not assigned.no ontology
